# Impacts of global change on the phyllosphere microbiome

**DOI:** 10.1111/nph.17928

**Published:** 2022-01-06

**Authors:** Yong‐Guan Zhu, Chao Xiong, Zhong Wei, Qing‐Lin Chen, Bin Ma, Shu‐Yi‐Dan Zhou, Jiaqi Tan, Li‐Mei Zhang, Hui‐Ling Cui, Gui‐Lan Duan

**Affiliations:** ^1^ Key Laboratory of Urban Environment and Health Institute of Urban Environment Chinese Academy of Sciences Xiamen 361021 China; ^2^ State Key Laboratory of Urban and Regional Ecology Research Center for Eco‐Environmental Sciences Chinese Academy of Sciences Beijing 100085 China; ^3^ Key Laboratory of Plant Immunity Jiangsu Provincial Key Laboratory for Organic Solid Waste Utilization Jiangsu Collaborative Innovation Center for Solid Organic Waste Resource Utilization National Engineering Research Center for Organic‐Based Fertilizers Nanjing Agricultural University Weigang, Nanjing 210095 China; ^4^ Faculty of Veterinary and Agricultural Sciences The University of Melbourne Parkville Vic 3010 Australia; ^5^ Zhejiang Provincial Key Laboratory of Agricultural Resources and Environment College of Environmental and Natural Resource Sciences Zhejiang University Hangzhou 310058 China; ^6^ Hangzhou Innovation Center Zhejiang University Hangzhou 311200 China; ^7^ Department of Biological Sciences Louisiana State University Baton Rouge LA 70803 USA

**Keywords:** climate change, microbes, one health, phyllosphere, plant microbiome, plant performance

## Abstract

Plants form complex interaction networks with diverse microbiomes in the environment, and the intricate interplay between plants and their associated microbiomes can greatly influence ecosystem processes and functions. The phyllosphere, the aerial part of the plant, provides a unique habitat for diverse microbes, and in return the phyllosphere microbiome greatly affects plant performance. As an open system, the phyllosphere is subjected to environmental perturbations, including global change, which will impact the crosstalk between plants and their microbiomes. In this review, we aim to provide a synthesis of current knowledge of the complex interactions between plants and the phyllosphere microbiome under global changes and to identify future priority areas of research on this topic.

## Introduction

The interactions between plants and their associated microbiomes are crucial for host performance and resilience to environment perturbations (e.g. global change) (Bulgarelli *et al*., 2013; Trivedi *et al*., 2020). Historically, plant microbiome research has focused mainly on the rhizosphere, including the symbiotic relationship between plant roots and bacteria and fungi, as well as soil‐borne pathogen dynamics. In the last decade or so, with the advent of molecular and genomic technologies, plant microbiome research has expanded rapidly, from the rhizosphere to phyllosphere, endosphere and seeds/fruits (Delmotte *et al*., 2009; Shade *et al*., 2017; Carrión *et al*., 2019; Grady *et al*., 2019). The phyllosphere represents the aboveground part of a plant, harbouring diverse microbes in both epiphytic (an organism that grows on the surface of a plant) and endophytic (an organism that lives within a plant) niches (Vorholt, 2012). When considering the upper and lower leaf surfaces, the total area of the phyllosphere on Earth is estimated to be over 10^9^ km^2^ and harbours up to 10^26^ bacterial cells (Lindow & Brandl, 2003; Vorholt, 2012; Penuelas & Terradas, 2014). The Earth and its ecosystems are undergoing rapid global changes such as climate change (e.g. warming and drought) and land‐use change (e.g. habitat loss and chemical fertilization), which are exerting pervasive impacts on ecosystem processes and functions, and various interactions among plants, microbes and the environment (Vitousek, 1994; Jansson & Hofmockel, 2019; Z. Zhou *et al*., 2020). A systematic understanding of how global change affects phyllosphere microbiomes could provide an important baseline for harnessing microbiomes to promote ecosystem resilience and plant productivity in a sustainable way. In this review, we aim to provide an overview of how global change will influence the complex interplay between the phyllosphere and its associated microbiomes and identify some priority areas for future research.

## Ecological functions of the phyllosphere microbiome

**Fig. 1:**
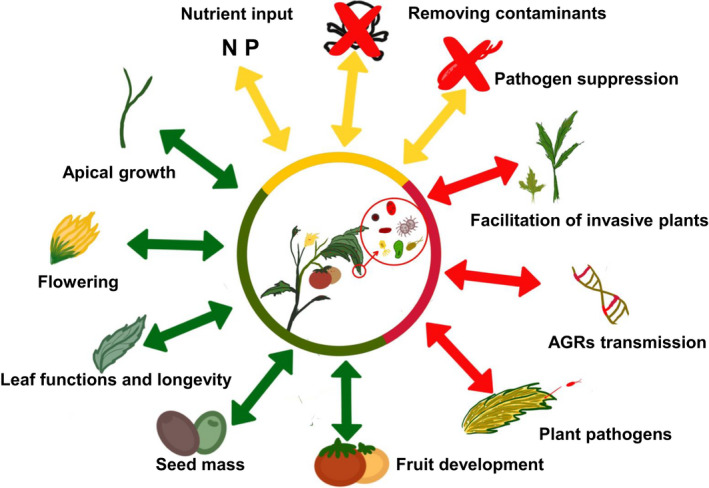
Ecological functions of the phyllosphere microbiome.

Phyllosphere‐colonizing microbes play critical roles in multiple functions (Fig. 1), including plant productivity and fitness, by affecting leaf functions and longevity, seed mass, apical growth, flowering and fruit development, and also play key roles in removing contaminants (Stone *et al*., 2018; Thapa & Prasanna, 2018; Liu *et al*., 2020). For instance, some plant growth‐promoting bacteria inhabiting the phyllosphere such as *Microbacterium*, *Stenotrophomonas* and *Methylobacterium* can improve the growth and nutritional status of the host plant by producing natural growth regulators (e.g. IAA) and fixing nitrogen (Madhaiyan *et al*., 2015; Abadi *et al*., 2020). The phyllosphere microbiome also plays important roles in reducing plant methanol (e.g. methylotrophs) and isoprene (e.g. isoprene‐degrading bacteria of the genus *Variovorax*) emissions to the atmosphere (Abanda‐Nkpwatt *et al*., 2006; Crombie *et al*., 2018). Moreover, the phyllosphere microbiome can play vital roles in maintaining plant health and suppressing the overgrowth of plant pathogens. For example, the phyllosphere microbiome can protect *Arabidopsis* plants against fungal pathogens and dysbiosis (a disruption to the microbiota homeostasis) that could have deleterious impacts on the host health (Ritpitakphong *et al*., 2016; Chen *et al*., 2020). Recent findings demonstrate that bacteria and yeasts colonizing nectar can modulate nectar chemical composition and consequently influence visitation/foraging by insect pollinators (Liu *et al*., 2019). As such, the phyllosphere microbiome contributes to the gut microbiome of insect pollinators and therefore influences their fitness and behaviour (Liu *et al*., 2019). Nonetheless, it should also be noted that phyllosphere microorganisms can have negative effects on host plants. The presence of a large and varied microbial community in the phyllosphere might increase competition with plants for nutrients and water (Saikkonen *et al*., 2015; Vacher *et al*., 2016). Some members of the phyllosphere microbiome might act as plant pathogens, resulting in different forms of plant disease (Lindow & Leveau, 2002; Whipps *et al*., 2008; Baker *et al*., 2010). Recently, Zhou *et al*. (2021b, 2021) reported that the phyllosphere microbiome is involved in the transmission of antibiotic resistance genes in the urban green facade. Another report (Bárta *et al*., 2021) suggests that the phyllosphere microbiome aids in the establishment of the invasive macrophyte *Hydrilla verticillata* L. under conditions of nitrogen scarcity. Increasing evidence shows that global change has pervasive impacts on plant health and ecosystem functioning, and harnessing the beneficial functions provided by the phyllosphere microbiome to enhance plant growth and fitness to face such impacts is considered a viable sustainable approach.

## Drivers and sources of the phyllosphere microbiome

As the phyllosphere is an open system, its associated microbiomes can come from multiple sources. The assembly of the phyllosphere microbiome is subject to: complex and variable environmental conditions (e.g. temperature, solar radiation, humidity, soil type and agricultural activity) (Vorholt, 2012; Aydogan *et al*., 2018; Truchado *et al*., 2019; Zhou *et al*., 2019; Stone & Jackson, 2021); plant species and genotypes (Singh *et al*., 2018; Schlechter *et al*., 2019; Wagner *et al*., 2020); the adaptability to particular foliar structures or resource secretions (e.g. leaf age and surface roughness, primary and secondary metabolites) (Crombie *et al*., 2018; Namdar *et al*., 2019; Sun *et al*., 2019); and the complex interactions between multiple trophic levels, such as microbe–microbe interactions and plant–herbivore–microbiome interactions (Remus‐Emsermann *et al*., 2013; Agler *et al*., 2016; Helfrich *et al*., 2018; Carlström *et al*., 2019; Liu *et al*., 2021). In addition, invasive plants caused by global change may influence the phyllosphere microbiome by altering soil properties and microbial communities and plant–soil feedback (McLeod *et al*., 2021). Phyllosphere microbiome composition is believed to be closely related to the surrounding environment of host plants, such as soil, air and nearby plant (Brown *et al*., 2020; Bell *et al*., 2021; Bernard *et al*., 2021). For example, soil microbes may enter the root tissues from emerging roots or wounds and constitute the root microbiota (Singh *et al*., 2020a), and part of this microbiota can be transferred to the aerial part of plants (i.e. phyllosphere) through xylem and phloem systems (Bell *et al*., 2021). This could partially explain the observations of microbial overlap between plant tissues and soil (Bai *et al*., 2015; Zarraonaindia *et al*., 2015; Chen *et al*., 2017; Xu *et al*., 2018). Furthermore, opening of leaf stomata and wounds provides a pathway for the transformation and migration between endophytes and epiphytes, and the opportunity for external microbes from aerosols and insects to colonize the plant, which also suggests that plants and the environment are interconnected (Mullens & Jamann, 2021; Xiang *et al*., 2021). Nevertheless, a recent study analysed the sources of phyllosphere microbes through a customized microcosm that was able to control external microbes (Zhou *et al*., 2021a, 2021), and showed that microbial sources from soil and air were limited. In another study, oak seeds were found to transmit a large part of microbes to roots and the phyllosphere, emphasizing that plant seeds are the reservoir of the plant microbiome (Abdelfattah *et al*., 2021), particularly in the early stages of plant growth (Berg & Raaijmakers, 2018; X. Zhou *et al*., 2020). Seeds can carry highly diverse and beneficial bacterial taxa to ensure the establishment of an optimal bacterial symbiosis for offspring (Liang *et al*., 2021). These studies highlight that inheritance of plant microbes may play a dominant role in shaping the phyllosphere microbiota.

Overall, the sources of phyllosphere microbes are complex and dynamic, influenced by both intrinsic plant factors and environmental conditions, while biotic and abiotic selection pressures must also be considered (Eldridge *et al*., 2021). A probable mechanism, therefore, is that the combination of environmental and genetic factors determines the assembly of microbial communities (Shakir *et al*., 2021). Uncovering how global change affects microbiome assembly, sources of transmission and plant–microbiome interactions in the phyllosphere could provide a mechanistic understanding for future microbiome manipulation.

## Hotspots and frontier trends in the phyllosphere microbiome responses to global change

**Fig. 2:**
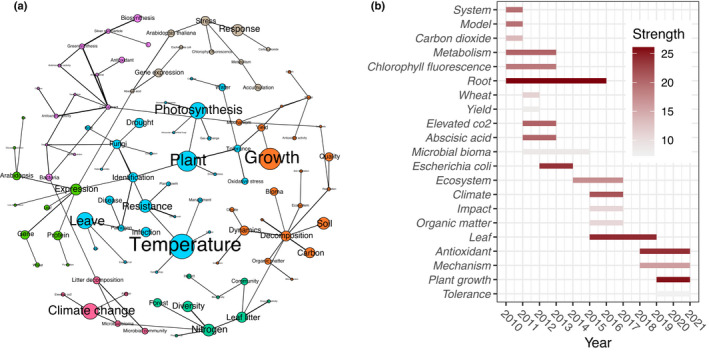
Bibliometric analysis of phyllosphere microbiome research based on the Web of Science Core Collection database from January 2010 to May 2021. (a) Keyword cooccurrence network. Nodes represent unique keywords; node size is proportional to the number of references; node colours indicate modules. (b) Burst word detection analysis. Length represents the burst status duration; colour saturation indicates citation burst strength. Bibliometric analysis was conducted by retrieving citation data based on a topic search using as query: ‘N deposition OR nitrogen deposition OR CO2 OR carbon dioxide OR precipitation OR temperature OR climate change) AND (phyllospher* OR leaf OR leaves) AND (fung* OR bacteria* OR microb* OR archaea* OR virus OR viral OR protist*’. The results were filtered to include items from January 2010 to May 2021, and were further analysed by CiteSpace (Chen *et al*., 2010) to highlight the hotspots and frontier trends in the phyllosphere microbiome responses to global change.

Bibliometric analysis was conducted by retrieving citation data from the Web of Science Core Collection database to highlight the hotspots and frontier trends in the phyllosphere microbiome responses to global change. Keyword cooccurrence network analysis showed that recent research has focused mainly on the relationships between phyllosphere microbiomes and plant growth and health under global change scenarios. These relationships also include interactions between pathogenic bacteria and plant pathogen resistance (Fig. 2a). Phyllosphere microbiomes are faced with increased stress caused by climate change, particularly by warming and drought. Climate change stresses may result in unstable states of microbial communities, wherein a reduction of beneficial taxa weakens plant resistance to pathogen invasion and disease development. Furthermore, phyllosphere microbiomes also participate in carbon and nitrogen cycling by engaging in nitrogen fixing, metabolizing plant metabolites and producing volatile organic compounds (Madhaiyan *et al*., 2015; Farre‐Armengol *et al*., 2016; Cernava *et al*., 2019); how carbon and nitrogen cycling mediated by the phyllosphere microbiome respond to climate change have not clearly determined. Although network analysis indicates that the model plant *Arabidopsis thaliana* is the most popular research taxon, a new plant model that is more agriculturally relevant is urgently needed to study crop–microbiome interactions for the development of effective microbiome tools for sustainable agriculture. Burst word detection analysis was further used to show a time‐series pattern of keywords, exploring research trends and advances in phyllosphere microbial ecology studies in response to global change over the last decade (Fig. 2b). Plant growth‐ and stress tolerance‐related research has seen the greatest increase since 2018 and therefore represents the current hotspot. A methodological driver for the recent increase in mechanism‐related research may be that the development of emerging technologies has allowed researchers to disentangle the mechanisms of the phyllosphere microbiome regulating plant growth and tolerance.

## Impacts of agricultural fertilization on the phyllosphere microbiome

Modern agricultural production relies heavily on the use of chemical fertilizers, such as nitrogen (N), phosphorus (P) and potassium (K) fertilizers. Global agricultural production is expected to increase by 70% by 2050 to feed the increasing human population (Singh *et al*., 2020b; Haskett *et al*., 2021), and the use of chemical fertilizers is likely to increase significantly in future agricultural production. However, intensive fertilization could cause soil degradation such as acidification and environmental pollution (Raza *et al*., 2020). Currently, most studies on the impacts of chemical fertilization on microbial communities have focused on soil and the rhizosphere, while there is a paucity of studies investigating how phyllosphere microbiomes respond to chemical fertilization (Hartman *et al*., 2018; Trivedi *et al*., 2020). In general, the phyllosphere harboured a less diverse microbial population including bacteria, fungi and protists than soil and rhizosphere, while the alpha‐ and beta‐diversity of these phyllosphere‐associated microbes often showed more resistance to fertilization (Sun *et al*., 2021a,b). One explanation for this could be the open nature of the phyllosphere, as phyllosphere‐associated microbes are influenced by multiple factors within dynamic and heterogeneous environments (Lindow & Brandl, 2003; Remus‐Emsermann & Schlechter, 2018), which may weaken the influence of fertilization regimes on phyllosphere microbial variations. In addition, a recent study focusing on the soil–plant continuum of maize, wheat and barley has demonstrated host selection plays a more important role in shaping phyllosphere assembly and network complexity than fertilization practices (Xiong *et al*., 2021b). Nevertheless, fertilization process may influence some specific microbial taxa in the phyllosphere. For instance, excessive application of chemical N fertilizer increased the relative abundance of potential fungal plant pathogens in the leaf endosphere (Xiong *et al*., 2021a). Similarly, a study on sorghum showed that long‐term fertilization regimes did not significantly influence the diversity and composition of protistan communities in the phyllosphere, but some protistan consumers (e.g. Amoebozoa) were significantly influenced by fertilization (Sun *et al*., 2021b). Additionally, other macro‐ and micronutrients also play a role in phyllosphere microbiome assembly. For example, application of these nutrients in soil increased microbial biodiversity but reduced the relative abundance of pathogen *Candidatus* Liberibacter asiaticus (CLas) in the phyllosphere of Gannan Navel Orange (Y. Zhou *et al*., 2021b, 2021). Although these studies provided valuable information, the fundamental knowledge of the mechanisms underlying phyllosphere microbiome assembly and activity under fertilization remains in its infancy.

In addition to fertilization, microbial communities in agricultural ecosystems are usually influenced by agronomic management regimes (e.g. organic and conventional management). It has beenreported that organic farming increased fungal alpha diversity in the wheat phyllosphere, compared with conventional management (Karlsson *et al*., 2017). A recent study also suggested that agricultural management (i.e. organic, transition and conventional) strongly influenced the composition, functions and cooccurrence networks of the sugarcane phyllosphere microbiome (Khoiri *et al*., 2021). Organic farming was associated with a complex microbial network and enriched some plant growth‐promoting bacteria such as *Bradyrhizobium* and *Bacillus*, whereas conventional practice decreased the abundance of functional genes involved in cell motility and energy metabolism of phyllosphere microbiomes (Khoiri *et al*., 2021). Emerging evidence indicates that agricultural management is an important factor driving phyllosphere microbiome assembly. Uncovering phyllosphere–microbiome interactions and their molecular mechanisms under different agricultural management practices can provide new scientific knowledge to harness the phyllosphere microbiome for plant productivity and sustainable agriculture.

## Impacts of global warming on the phyllosphere microbiome

**Fig. 3:**
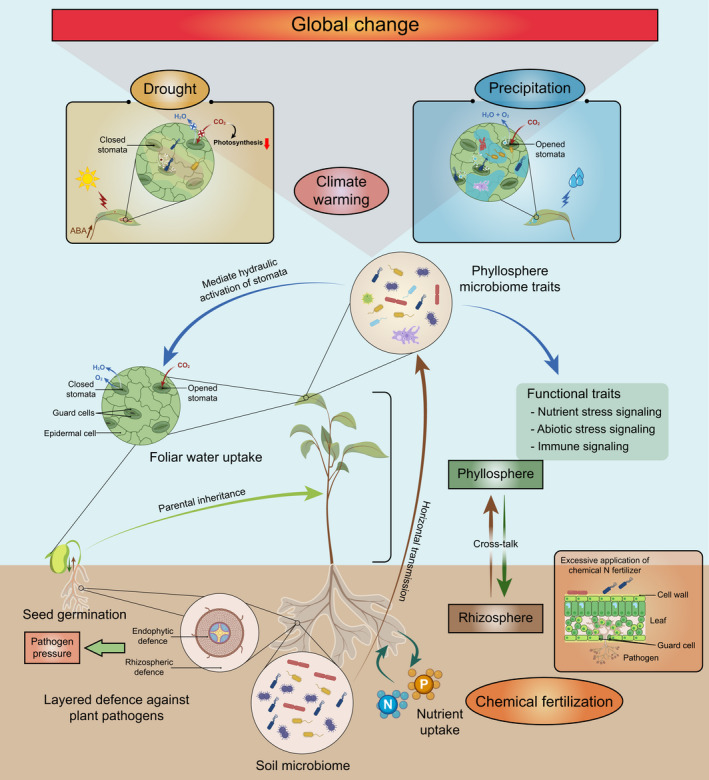
Impact factors of the phyllosphere microbiome. The phyllosphere comprises the aboveground part of a plant, harbouring diverse microbes in both epiphytic and endophytic niches. These microbiomes derive from vertical transmission via parental inheritance, and horizontal transmission by surrounding environments (e.g. soil and air). On the one hand, global changes such as climate warming, drought and precipitation might impact leaf functional traits and phyllosphere microbiome traits, and the latter mediates the hydraulic activation of stomata relevant to the pathway for foliar water uptake. On the other hand, chemical fertilization can also affect the phyllosphere microbiome by changing rhizosphere communities and leaf morphology.

Global warming caused by the ‘greenhouse effect’ is predicted to have major consequences on element cycling and the functioning of terrestrial ecosystems such as vegetation dynamics (Vitousek, 1994; Jones *et al*., 1998; Norby & Luo, 2004), which will substantially impact the phyllosphere microbiome (Zhu & Penuelas, 2020) (Fig. 3). Based on the Intergovernmental Panel on Climate Change (IPCC, 2014), global mean surface temperature is estimated to increase by 2–3°C within the next few decades (Stocker, 2014), which is predicted to result in a global increase in drought frequency and duration.

In recent decades, experimental studies of climate warming effects have focused mostly on the soil microbiome (Yergeau *et al*., 2012; Jansson & Hofmockel, 2019; Z. Zhou *et al*., 2020), while the potential impacts of warming on the abundance and compositions of the phyllosphere microbiome have largely been overlooked and are only just beginning to be studied. For example, based on a long‐term field warming experiment on a grassland (dominated by *Arrhenatherum elatius* and *Galium album*) with increasing surface temperature of 2°C, Aydogan *et al*. (2018, 2020) found that warming does not affect the total colonization and the concentration of leaf‐associated bacterial cells but shifted the diversity and phylogenetic composition of the bacterial communities. More importantly, warming‐induced decreases of beneficial bacteria (e.g. *Sphingomona*s spp. and *Rhizobium* spp.) and enhancement of potentially pathogenic bacteria (e.g. Enterobacteriaceae, *Pseudomonas*, and *Acinetobacter*) in the phyllosphere may indicate that warming increases the potential transmission of pathogens in grassland ecosystems (Aydogan *et al*., 2018). In addition to affecting phyllosphere bacterial communities, climate warming also decreased fungal richness, reduced evenness and shifted the overall fungal community composition on pedunculate oak (*Quercus robur*) (Faticov *et al*., 2021) and a boreal forest tree (*Populus balsamifera* L.) (Balint *et al*., 2015). In contrast to potential bacterial pathogens (e.g. *Acinetobacter*), warming negatively affected putative fungal pathogens (Aydogan *et al*., 2018; Faticov *et al*., 2021). These elegant studies provided valuable information on the impacts of climate warming on the profiles of the phyllosphere microbiome. However, care must be taken when interpreting the outcomes of each study, as only limited plant species and genotypes have been considered. The differences in leaf traits, nutrient content and primary/secondary metabolites among genotypes could result in differential colonization of microorganisms, which may mask the effects of climate warming (Wagner *et al*., 2016). For instance, in contrast to the above observation, other studies have indicated that long‐term warming experiments caused no significant changes in the foliar fungal community composition of three perennial grass species (*Achnatherum lettermanii*, *Festuca thurberi* and *Poa pratensis*) (Kazenel *et al*., 2019; Kivlin *et al*., 2019). Thus, it is unclear whether the response of the phyllosphere microbiome to climate warming is consistent across plant species and genotypes, although some excellent studies have been conducted on this topic (Faticov *et al*., 2021). Moreover, the present investigations on phyllosphere microbiomes have focused generally on bacterial and fungal communities, while little attention has been paid to other microbes such as archaea and protists. All these observations have highlighted the need to improve our understanding of climate warming on the phyllosphere microbiome.

## Impacts of precipitation and drought on the phyllosphere microbiome

Precipitation has begun to show a long‐term downward trend under global climate change, resulting in a global increase in drought frequency and duration (Sardans *et al*., 2008). Meanwhile, extreme weather events including floods and drought are being recorded more acutely and frequently. Such a change at the global scale is expected to have significant impacts on global agricultural production, as it can influence plant growth and plant disease occurrence by altering humidity and water availability (Howden *et al*., 2007; Xin *et al*., 2016; Xu *et al*., 2021; Romero *et al*., 2022). A recent large‐scale survey suggested that precipitation is the most important predictor of fungal communities and the abundance of fungal plant pathogens, and the authors suggested that the abundance of fungal plant pathogens could increase by up to 100‐fold by 2050, especially in coastal regions (Chen *et al*., 2021). The interactions between water status, soil fertility and arbuscular mycorrhizal fungi could also shift the phyllosphere microbiome; for example, both water status and mycorrhizal disruption could reduce phyllosphere bacterial richness, with a more homogeneous bacterial community composition of the tomato (*Solanum lycopersicum*) phyllosphere (Debray *et al*., 2022). In addition, it was found that high humidity can transform nonpathogenic *Pseudomonas syringae* strains into virulent pathogens and induce dyshomeostasis of the commensal bacterial community in the phyllosphere by affecting water status inside the apoplast (Xin *et al*., 2016). Furthermore, drought stress not only affects phyllosphere microbial compositions (Bechtold *et al*., 2021) but also community assembly processes. A recent study on sorghum systems with abundant sampling indicated that the assembly of phyllosphere mycobiomes was determined by stochastic processes (e.g. drift or stochastic dispersal) in the early stage of host development when sorghum is drought‐stressed (Gao *et al*., 2020). Regarding precipitation, recent work on the wetland macrophyte broadleaf cattail (*Typha latifolia*) showed that rain events did not have a significant effect on the richness or evenness of its phyllosphere bacterial community (Stone & Jackson, 2021). By contrast, climatic and leaf‐related variables effectively shaped seasonal dynamics in phyllosphere diversity and composition (Stone & Jackson, 2021). Under the scenario of climate change, improving our understanding of how plant species and their microbiomes can cope with drought events is one of the most relevant topics in plant science. Foliar water uptake (FWU) has been identified as a mechanism commonly adopted by trees and other plants from various biomes and could be used to predict the sensitivity of plant species to drought (Schreel & Steppe, 2020). In addition to morphological and anatomical traits and leaf age (Schreel & Steppe, 2020), leaf wettability also depends on the degree of cover by the phyllosphere microbiome (epiphytic and endophytic organisms) and thus affects their hydrophobicity (Rosado & Almeida, 2020). For example, the cuticular permeability that allows the diffusion of water through the cuticle might be increased by biosurfactants produced by epiphytic bacteria (Park *et al*., 2018), indicating their potential effect on FWU. Moreover, the phyllosphere microbiome can also mediate the hydraulic activation of stomata, which is relevant to the pathway for FWU. For example, fungal leaf endophytes may increase stomatal conductance, while bacteria may mediate stomatal closure and opening (Friesen *et al*., 2011). Considering the stomata as the gateway for the entrance of pathogens to plants (Gudesblat *et al*., 2009), regulation of the stomatal aperture by the phyllosphere microbiome is also a mechanism associated with plant defence. All these observations suggest that phyllosphere‐associated microbiomes have great potential to improve plant resistance to future drought (Rolli *et al*., 2015; Llorens *et al*., 2019; de Vries *et al*., 2020; Xu *et al*., 2021).

## Ecoevolutionary dynamics between the phyllosphere and its microbiomes under global climate change

The ecoevolutionary dynamics of plant–microbiome symbiosis systems are of increasing interest. Unfortunately, little has been done regarding the phyllosphere. Among current studies, the impacts of plant evolutionary history and contemporary evolution on plant, soil and rhizosphere microbiome responses to climate change have received much attention (Lambers *et al*., 2009; Fitzpatrick *et al*., 2020; Petipas *et al*., 2021).

As the outcome of past evolutionary history, the phylogenetic relationship between plant species has been found to be able to interact with climate change to modify plant microbiomes (Naylor *et al*., 2017). In the absence of drought, cereal grass phylogeny was shown to determine rhizosphere microbiome composition. Such effects of host evolutionary history on microbiomes have been widely observed in plants, especially those with agricultural significance (Stolf‐Moreira *et al*., 2011; Peiffer *et al*., 2013; Santos‐Medellín *et al*., 2017). Drought, however, promoted the abundance of Actinobacteria, weakening the importance of host evolutionary history for microbiome community structure (Naylor *et al*., 2017). In addition, evolution at the contemporary timescale, in both microbiomes and plants, can mediate plant–microbiome responses to climate change. Microbes often have a large population size and high genetic variation, which translate to strong evolutionary dynamics to influence ecological processes (Yoshida *et al*., 2003; Frantzeskakis *et al*., 2020). For example, using the synthetic community approach, Batstone *et al*. (2020) showed that rapid evolution has occurred in the nodule‐forming bacterium *Ensifer meliloti* and promoted mutualism between the bacterium and the plant host *Medicago truncatula*. Unlike microbes, more barriers exist for plant evolution. Nevertheless, recent evidence has suggested the possibility of rapid evolution in plants. In an experiment by terHorst *et al*. (2014), adaptation was found to occur in *Brassica rapa* populations after a three‐generation drought treatment. When transplanted into a common garden under ambient wet conditions, *B. rapa* populations (adapted vs unadapted to drought) showed different abilities in shaping the soil microbiomes.

We recognize that most existing work has focused on the ecoevolutionary dynamics between plants and soil/rhizosphere microbiomes, not microbiomes in other plant compartments, including the phyllosphere. Also, little attention has been given to several evolutionary processes unique to plants, such as within‐ and cross‐species hybridization (Rieseberg & Carney, 1998) and the emergence of polyploidy plants (Adams & Wendel, 2005), which can introduce novel genetic variation to wild plant populations. Recent progress in research on ecoevolutionary dynamics has prompted the development of new model systems for study of the role of plant evolution in ecological processes (Williams *et al*., 2016; Hart *et al*., 2019). For example, there is increasing interest in using aquatic floating plants of the family Lemnaceae (commonly known as duckweeds) to examine plant ecological and evolutionary responses to environmental change (Armitage & Jones, 2019; O'Brien *et al*., 2020). Duckweeds have high within‐species genotypic and phenotypic diversity (Hart *et al*., 2019; O'Brien *et al*., 2020), making it possible to observe the changes in both species composition (ecological dynamics) and genotypic composition (evolutionary dynamics) during plant–microbiome interactions at the same timescale. The high tractability of these model systems can extend the scope of observational studies, providing more mechanistic understanding of the importance of evolution in plant–microbiome systems.

## Conclusions and perspectives

The phyllosphere microbiome plays an essential role in increasing the ability of a plant to pass through environmental filters. Currently, however, we have limited capacity to predict the consequences of the shift in the phyllosphere microbiome for ecosystem functioning under a changing environment. Some fundamental questions remain largely unresolved: (1) What are the mechanisms within and across plant hosts that underpin host–microbe interactions? (2) What are the major microbial taxa in the phyllosphere that control or mediate plant performance (e.g. nutrient uptake, plant disease suppression or growth)? (3) How does the phyllosphere microbiome interact with other plant microbiomes? (4) How can we manage the phyllosphere microbiome to increase plant health and performance in a changing world? (5) How will host and the phyllosphere microbiome evolve in response to global changes, and what impacts will this ecoevolution have on ecosystem functions? Therefore, we argue that advancing our fundamental understanding of the impacts of global changes on the phyllosphere microbiome and related ecosystem functions requires interdisciplinary investigations. We need to shift the focus from the level of community ecology to ecosystem ecology for a better understanding of the mechanisms underlying responses of the ‘holobiont’ (assemblage of a plant and its microbiome living in or around it) to global changes. A systems approach is needed to understand the complex interactions between the phyllosphere microbiome and host fitness, and the ecological functions of these microbes for plant nutrition uptake, growth and survival under global climate change.
